# Interactions of the Melanocortin-4 Receptor with the Peptide Agonist NDP-MSH

**DOI:** 10.1016/j.jmb.2010.06.028

**Published:** 2010-08-20

**Authors:** Kathryn L. Chapman, Gemma K. Kinsella, Alan Cox, Dan Donnelly, John B.C. Findlay

**Affiliations:** 1 School of Biochemistry and Molecular Biology, University of Leeds, Leeds LS2 9JT, UK; 2 Department of Biology, National University of Ireland Maynooth, Maynooth, Co. Kildare, Ireland

**Keywords:** MC4R, melanocortin-4 receptor, NDP-MSH, [Nle_4_,d-Phe_7_]melanocyte-stimulating hormone, TM, transmembrane, MCR, melanocortin receptor, GPCR, G-protein-coupled receptor, MSH, melanocyte-stimulating hormone, MTII, melanotan II, d-Nal, β-(2-naphthyl)-d-alanine, ECL, extracellular loop, hMC4R, human MC4R, PDB, Protein Data Bank, WT, wild type, CRE, cyclic AMP response element, CuP, copper 1,10-phenanthroline, MC1R, melanocortin-1 receptor, PDF, probability density function, comparative model, cysteine cross-linking, G-protein-coupled receptor (GPCR), melanocortin-4 receptor (MC4R), [Nle_4_,d-Phe_7_]melanocyte-stimulating hormone (NDP-MSH)

## Abstract

Melanocortin-4 receptor (MC4R) has an important regulatory role in energy homeostasis and food intake. Peptide agonists of the MC4R are characterized by the conserved sequence His_6_-Phe_7_-Arg_8_-Trp_9_, which is crucial for their interaction with the receptor. This investigation utilized the covalent attachment approach to identify receptor residues in close proximity to the bound ligand [Nle_4_,d-Phe_7_]melanocyte-stimulating hormone (NDP-MSH), thereby differentiating between residues directly involved in ligand binding and those mutations that compromise ligand binding by inducing conformational changes in the receptor. Also, recent X-ray structures of G-protein-coupled receptors were utilized to refine a model of human MC4R in the active state (R^⁎^), which was used to generate a better understanding of the binding mode of the ligand NDP-MSH at the atomic level.

The mutation of residues in the human MC4R—such as Leu106 of extracellular loop 1, and Asp122, Ile125, and Asp126 of transmembrane (TM) helix 3, His264 (TM6), and Met292 (TM7)—to Cys residues produced definitive indications of proximity to the side chains of residues in the core region of the peptide ligand. Of particular interest was the contact between d-Phe_7_ on the ligand and Ile125 of TM3 on the MC4R. Additionally, Met292 (TM7) equivalent to Lys(7.45) (Ballesteros numbering scheme) involved in covalently attaching retinal in rhodopsin is shown to be in close proximity to Trp_9_.

For the first time, the interactions between the terminal regions of NDP-MSH and the receptor are described. The amino-terminus appears to be adjacent to a series of hydrophilic residues with novel interactions at Cys196 (TM5) and Asp189 (extracellular loop 2). These interactions are reminiscent of sequential ligand binding exhibited by the β_2_-adrenergic receptor, with the former interaction being equivalent to the known interaction involving Ser204 of the β_2_-adrenergic receptor.

## Introduction

Melanocortin receptors (MCRs) belong to the G-protein-coupled receptor (GPCR) family A and, upon stimulation, elevate cyclic adenosine-3′,5′-monophosphate (cAMP) levels. The melanocortin-4 receptor (MC4R) is one of five members of the MCR family and is located primarily in the brain, where it has an important role in energy homeostasis and regulation of food intake. The MC4R is, therefore, a potential therapeutic target in both anorexia and obesity.^1–3^ The agonists of the receptor, the melanocyte-stimulating hormones (MSHs) (or α-MSH, β-MSH, and γ-MSH), are derived from proopiomelanocortin by proteolysis, and all have a conserved core tetrapeptide sequence His_6_-Phe_7_-Arg_8_-Trp_9_,^4–8^ which is the minimal sequence required for activation of the receptor.^9,10^

An important secondary structural element found in peptide hormones that stimulate GPCRs is a reverse turn, or β-turn, where the direction of the peptide chain is reversed at the fourth residue.^4,11^ The distance between the first residue and the fourth residue is defined as less than 7 Å, and an α-helical secondary structure is not formed. A hydrogen bond between the carbonyl group of the first residue and the amide of the third peptide bond stabilizes β-turns within the peptide chain.^12^ Several NMR structures of a peptide agonist based on α-MSH-derived analogues^13–15^ and melanotan II (MTII)^13,16–21^ have been released. Due to the size and flexibility of the structures, a general consensus for the orientation of most of the amino acids is still being debated.^22^ However, there is general agreement that a β-turn-like structure forms around core residues 6–9, but even here, there are variations. The NMR studies of Cho *et al.* on [Nle_4_,d-Phe_7_]melanocyte-stimulating hormone (NDP-MSH) suggested that the central residues forming the turn conformation are the His_6_-d-Phe_7_ pair.^20^ In contrast, Hogan *et al.* studied cyclic decapeptides with the consensus tetrapeptide sequence His_6_-d-Phe_7_-Arg_8_-Trp_9_ locked in a type II′ β-turn, with the heart of the turn being d-Phe_7_-Arg_8_.^23^

Alanine scanning of the tetrapeptide demonstrated the functional consequence of modifying each residue, and evidence has revealed that amino acid substitution within this tetrapeptide sequence results in a dramatic decrease in affinity for the receptor. In contrast, replacement of the l-Phe residue for d-Phe at position 7 of α-MSH (NDP-MSH) potentiated the effect of the agonist.^7,24^ The core tetrapeptide sequence has been modified further into a cyclic compound known as MTII (see Supplementary Material). Enlarging the ring structure of MTII by substituting d-Phe for β-(2-naphthyl)-d-alanine (d-Nal), which is a bulkier amino acid, resulted in the production of the first high-affinity antagonist SHU9119^25^ (see Supplementary Material). Truncation studies with α-MSH suggested that terminal residues 1–3, 5, 10, and 12 played a role in potency, with the remainder having no significant role in potency or affinity.^26,27^ Furthermore, NDP-MSH residues 1–3, 5, and 13 have also been shown to be important in receptor response.^28^

Mutagenesis studies^6,23,29–39^ and computer modeling^13^ have predicted key residues contained within the MC4R that are responsible for interacting with agonist and antagonist ligands. Two types of interactions have been proposed: an ionic interaction between the positively charged Arg_8_ of the core tetrapeptide and one or more acidic residues on the receptor in transmembrane (TM) 2 (Glu100) and TM3 (Asp122 and Asp126), and hydrophobic contacts between the side chains of d-Phe_7_ and Trp_9_ on the ligand and the hydrophobic side chains on TM4 (Phe184 and Tyr187), TM6 (Phe261, Phe262, His264, and Phe267), and TM7 (Phe284).^34,35^ Mutations at residues Asp122 and Asp126 (TM3) of the receptor resulted in a large decrease in affinity for the agonist (containing Arg_8_), but not for the antagonist (with norleucine Nle_8_ substituted for Arg_8_). This suggested a direct ionic interaction between Arg_8_ and Asp122(3.25) and/or Asp126.^35,37^ Recent mutagenesis studies have focused on the binding pocket of the peptide agonists α-MSH,^40^ γ-MSH,^41^ and *N*-d-Nal(2′)^7^-adrenocorticotropic hormone(1–17)^44^ and on the binding pocket of nonpeptide agonists such as tetrahydroisoquinolone.^45^ However, many mutations in the MC4R reduce the affinity for an agonist that may not directly interact with the ligand, but actually vary the conformation of the receptor with a consequent change in the fine structure of the binding site. Residues proposed to control receptor conformation include Leu106 of extracellular loop (ECL) 1 and Ile125(3.28) (TM3).^23,37,42,43^

The aim of this work is to identify molecular interactions that occupy the binding pocket for the peptide agonist of the MC4R by covalently attaching cysteine peptide analogues of NDP-MSH to native Cys residues on the receptor through thiol chemistry. Contacts involving the core tetrapeptide of NDP-MSH are mapped when designated amino acids within the human MC4R (hMC4R) are substituted with a Cys residue and are shown to covalently cross-link to Cys-containing peptides within the tetrapeptide agonist sequence.

In an effort to deliver a more meaningful analysis, we performed our cross-linking work in conjunction with molecular modeling studies to provide a more detailed examination of potential binding site interactions. For many years, comparative modeling of class A GPCRs was based on inactive R-state (bovine rhodopsin) crystal structures, with an ongoing challenge being the development of R^⁎^ active-state models. Several reports have indicated that GPCR activation is accompanied by rigid-domain motion of the intracellular portion of TM6 relative to TM3^46,47^ and by counterclockwise rotations of TM6^48^ and TM3^49^ from an extracellular viewpoint. The crystal structure of opsin, solved in 2008, indicated that TM6 is tilted outwards by 6–7 Å at the intracellular end, while TM5 is closer to TM6 when compared with the R-state structures [Protein Data Bank (PDB) code 3CAP; 2.9 Å].^50^ More recently, Scheerer *et al.* published a crystal structure of the active GPCR opsin complexed with a C-terminal peptide derived from the Gα subunit of transducin GαCT(340–350)K341L (PDB code 3DQB; 3.2 Å), whose binding was further facilitated by a restructured TM7–helix 8 kink.^51^ In this study, we utilized these recently elucidated crystal structures to generate R^⁎^-state models of the MC4R from which a complex with NDP-MSH was developed in a ligand-based modeling approach. The model was utilized to examine the proposed interactions and the role of the various mutations studied here and reported earlier in the literature. In the process, we attempted to define a preferred structure of the bound ligand, most specifically the position of the putative β-turn.

## Results

### MC4R R⁎ model validation and analysis

Structural models of the hMC4R R^⁎^ state were obtained as described in Materials and Methods using the known 2.9–3.2 Å resolution structures of the homologous class A GPCR opsin.^50,51^ The validity of the developed hMC4R R^⁎^ models was examined using a number of tools. The best model, based on the 3DQB template with sequence alignment as depicted by Chai *et al.*, had only one stereochemical outlier (namely, Ser109 of ECL1) and was hence deemed stereochemically satisfactory.^29^ Additionally, the model had an initial ERRAT^52^ score of 71.386, which is greater than the cutoff at 50. After minimization of the model in MOE,^53^ a score of 91.167, which is comparable to 89.058 scored by the crystal structure and is indicative of a good ratio of nonbonded interactions, was obtained.

**Fig. 1:**
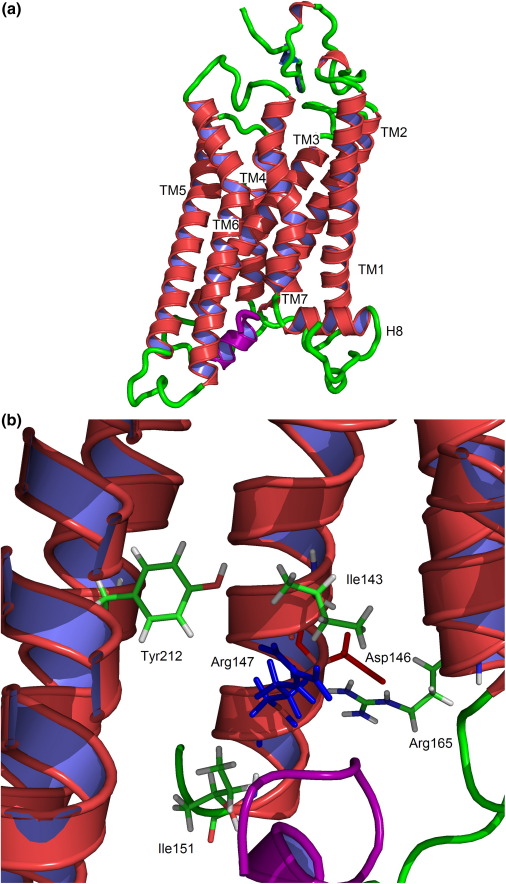

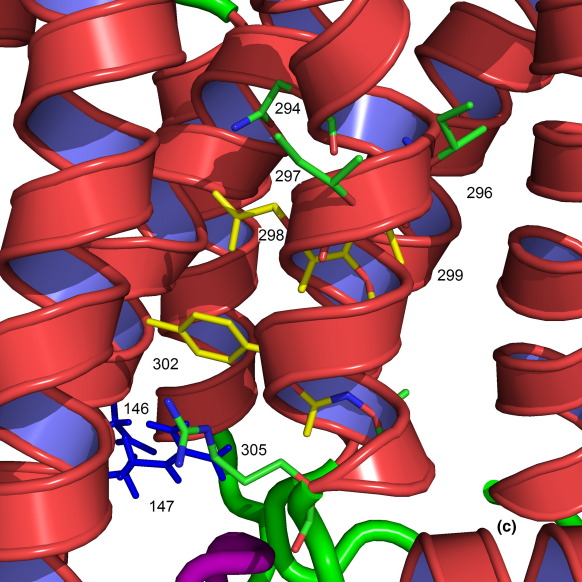
(a) Three-dimensional image of the R^⁎^ hMC4R model. GαCT(340–350)K341L) is indicated in purple. (b) Three-dimensional image zoomed to the area of the E/DRY motif, with interacting residues indicated. The outer helices were removed for clarity. (c) Three-dimensional image zoomed to the area of the NPxxY motif (in yellow); Asp146 and Arg147 are indicated in blue, with interacting residues identified (see the text for further details).The images were generated using PyMOL.^54^

An outline of the developed MC4R model showing the seven TM regions and the two proline kinks present at Pro260 (TM6) and Pro299 (TM7) is presented in Fig. 1. The N-terminus, C-terminus, and loops were modeled from the opsin template with subsequent loop refinement. Notably, the highly conserved ECL1 of the MCRs, containing the acidic Asp-x-Asp motif, interacts with the N-terminus and TM2. MCRs lack the characteristic long ECL2 loop of class A GPCRs at the entrance of the ligand binding pocket, which may allow greater access to the binding pocket.^54^ In the opsin crystal structure (PDB code 3DQB), GαCT(340–350)K341L binds to an intracellular site opened by an outward tilt of TM6, a pairing of TM5 and TM6, and a restructured TM7–helix 8 kink.^51^ A number of interactions with TM3, TM5, and TM6 of MC4R involving GαCT and our developed MC4R model were observed. First, His222 at the end of TM5 comes into contact with Ile340 and Glu342 of GαCT. From TM3, Thr150 and Arg147 of the conserved DRY motif form hydrogen bonds with Lys345 and Asp346 of GαCT, respectively, while Lys242 of TM6 hydrogen bonds with Phe350 of GαCT.

### MC4R R⁎ model: Analysis of conserved motifs

Next, we examined the roles of conserved motifs in both the opsin crystal structure and the MC4R R^⁎^ model that are postulated to have a role in activation. In our static hMC4R model, the Asp146-Arg147 interaction of the DRY motif is broken, with Asp146 forming a hydrogen bond with Arg165 at the start of TM4 (at 2.69 Å between the closest heavy atoms), while Arg147 hydrogen bonds with Ile143 (2.82 Å) and Ile151 (2.82 Å) of TM3 (Fig. 1). In both Ops^⁎^ and Ops^⁎^-GαCT structures, the hydroxyl group of Tyr223 from TM5 replaces the carboxyl group of Glu134 in its interaction with Arg135 of ERY (2.89 Å), with the Arg side chain then swinging into the center of the GαCT binding pocket to form the floor of the pocket. In our hMC4R model, the equivalent Tyr212 of TM5 is in close proximity to Arg147 (4.76 Å) and forms hydrogen bonds with Leu140 (2.55 Å) of TM3, and Met208 (2.95 Å) and Phe216 (2.81 Å) of TM5.

The highly conserved aspargine (N) of the NPxxY motif in TM7 has been postulated to act as an on/off switch by adopting two different conformations in the inactive and active states.^55^ In the inactive state, the Asn(7.49) side chain is orientated towards TM6, either via a water molecule in rhodopsin^56^ or through an interaction with the Thr(6.43)/Asp(6.44) motif in the glycoprotein hormone receptor family.^55^ Upon receptor activation, Asn(7.49) is proposed to adopt the trans conformation to interact with Asp(2.50) of the (N/S)LxxxD motif in TM2. However, in the opsin crystal structures, Asn302 (Asn(7.49) of the NPxxY motif) in TM7 forms a hydrogen-bond interaction with neighboring Tyr306. This interaction is broken in our hMC4R model, with Asp298 (DPxxY motif) forming interactions with nearby Asn294 (2.82 Å) instead. Finally, Asp90 of (N/S)LxxxD in TM2 forms a hydrogen bond with Leu86 (2.94 Å) and Ser94 (2.97 Å) of TM2, Ser132 (2.74 Å) and Ser136 (2.63 Å), and Ser295 (2.59 Å) of TM7, while the corresponding Asp83 of opsin hydrogen bonds to the equivalent TM2 residues only (Fig. 1).

As a consequence of the template selected and the above analysis, we believe that our MC4R model represents a good intermediate R^⁎^ state, which is appropriate for docking an agonist ligand.

### Cross-linking hMC4R Cys mutants to NDP-MSH peptide analogues

This study was carried out with 13 peptide analogues of NDP-MSH, which contain a Cys residue at each position of the peptide in turn and with the hMC4R containing Cys mutations. It was necessary to characterize the efficacy of these cysteine mutations. The pEC_50_ values (log of the ligand concentration that gave 50% of maximum activity) of peptide agonists were determined using ligand-dependent production of cAMP production, as monitored using induced luciferase expression in the presence of its substrate luciferin (see Materials and Methods). This assay gave a pEC_50_ value of 8.27 ± 0.27 (Table 1) for NDP-MSH with human wild-type (WT) MC4R. This is comparable with the literature values of 9^57^, 8.3,^58^ and 9.2^59^ using the same technology for NDP-MSH with human WT MC4R. The results demonstrated that all mutant peptides elicited a reduction in potency, as determined by the cyclic AMP response element (CRE)-Luciferase assay for activity with NDP-MSH compared to the WT receptor (*p* < 0.05; paired *t* test). The mutant MC4Rs L106C and I125C exhibited a 10-fold decrease in potency with NDP-MSH, whereas D126C and H264C displayed a > 1000-fold decrease in potency, and D122C and M292C were not stimulated at all by NDP-MSH (Table 1).

Interestingly, when functional studies were performed to obtain pEC_50_ values for the binding of the Cys-containing peptide analogues 6, 7L, 7D, 8, and 9 to the mutant receptors, most showed little or no activity after stimulation by the agonist NDP-MSH. Peptide 8, however, stimulated the L106C receptor to a greater extent than NDP-MSH such that the pEC_50_ value was similar to that for the binding of NDP-MSH to WT MC4R (see Supplementary Material). This may suggest stabilization of a significant interaction.

Cross-linking studies were performed between the MC4R-containing Cys mutations and 13 peptide analogues of NDP-MSH. Disulfide bond formation was induced by incubation in the presence of copper 1,10-phenanthroline (CuP), as described in Materials and Methods. The presence of disulfide bonds between the receptor and the ligand was demonstrated by detection of the biotin attached to the peptide ligand at a position close to the molecular mass of the MC4R on Western blot analyses of the cross-linked samples, resolved by SDS-PAGE.

**Fig. 2:**
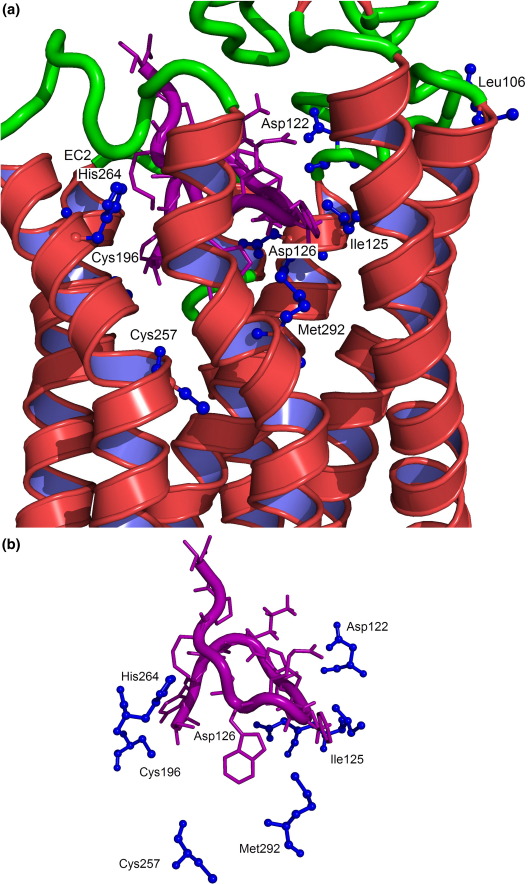

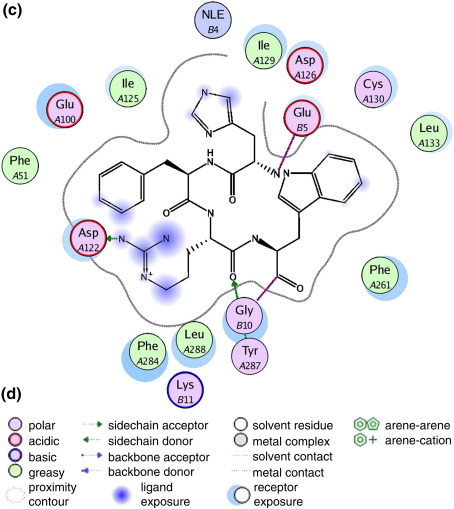
(a) Three-dimensional structure indicating relevant residues mentioned in this study: Leu106 (ECL1); Asp122, Ile125, and Asp126 (TM3); Cys196 (TM5); Cys257 and His264 (TM6); and Met292 (TM7). The position of the NDP-MSH peptide is presented in purple. A zoomed-in image of the region is presented in (b). The images were produced using PyMOL.^36^ (c) A MOE two-dimensional^53^ depiction of the interactions involving the core of the peptide (chain B) and the MC4R R^⁎^ model (chain A). The two-dimensional interaction caption is presented in (d).

**Fig. 3:**
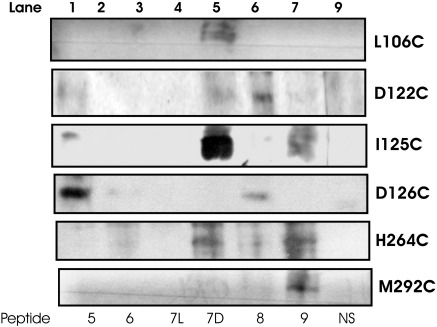
Interactions between peptide analogues and key residues in the binding pocket. Membrane samples prepared from HEK293 cells expressing the hMC4R with Cys mutations in key positions and incubated separately with 100 nM of each biotinylated cysteine-substituted NDP α-MSH peptide in the absence (T, total binding) or in the presence  (NS, nonspecific binding) of 50 μM SHU9119.

The key residues contained within the MC4R that were mutated to Cys include the tight acidic cluster in TM3 (Asp122 and Asp126) and the conserved family A residues (Leu106 and Ile125).^55^ Additionally, important residues believed to be located in the hydrophobic binding pocket on TM6 (His264), as well as the residue in the MC4R (Met292) that is equivalent to the residue involved in the binding of retinol to bovine rhodopsin (Lys296) (Fig. 2; Supplementary Material), were also mutated to Cys. Each of the aforementioned mutants was incubated with CuP in the presence of the NDP-MSH Cys peptide containing analogues 2, 5, 6, 7L, 7D, 8, 9, and 13 (Fig. 3). The degree of covalent cross-linking, as determined by Western blot analysis and chemiluminescence signal, is shown in Fig. 3. The intensity of the signal varies depending on the occupancy of the binding site by the biotinylated ligand. The results are discussed in detail below.

The core tetrapeptide of NDP-MSH (with both β-turn types d-Phe_7_-Arg_8_ and His_6_-d-Phe_7_) had been manually docked into the binding pocket of our MC4R model using Autodock^60^ and refined with MOE^53^ before the complete peptide was modeled *in situ* with Modeller9v5,^61^ as described in Materials and Methods. The modeled proximity of the residues in the peptide to the receptor and the experimental data were examined and compared (see Supplementary Material). In the process, it was found that the d-Phe_7_-Arg_8_ β-turn model for the peptide gave a better fit to the biochemical data and proximities. This is discussed in more detail below (Fig. 2).

### Interaction between Glu_5_ of NDP-MSH and MC4R

We examined the covalent interactions observed with the Cys-substituted core peptide residues and compared them to those predicted by our model. Peptide 5 was shown, through cross-linking, to interact with Asp122, Ile125, and, particularly, Asp126 (Fig. 3). Our molecular model is in agreement with the side chain of Glu_5_ being in close proximity to Asp126 of the receptor (C^α^–C^α^ distance of 5.8 Å in the model; closest distance of 2.6 Å). This was the most intense biotin-detected band observed by biochemical studies and predicted by computational studies. Additionally, Glu_5_ on the NDP-MSH peptide lies close to Asp122 (C^α^–C^α^ distances of 7.1 Å; closest distance of 2.5 Å) and Ile125 (C^α^–C^α^ distances of 8.0 Å; closest distance of 6.1 Å) of the MC4R. Furthermore, the model predicts a novel hydrogen bond between Ser127 (TM3) and Glu_5_ on the NDP-MSH peptide.

### Interactions between His_6_ of the tetrapeptide and MC4R

A band with a low signal was observed, via Western blot analysis, between peptide 6 (Cys/His substitution) and the residues Asp122, Asp126, and His264 on the receptor (Fig. 3). In our model, His_6_ interacts with d-Phe through arene–arene stacking and with Arg_8_ and Trp_9_ through backbone interactions. The cross-linking data indicated that peptide 6 interacts weakly with L106C (C^α^–C^α^ distance of 17.6 Å; shortest distance of 16.5 Å), D122C (C^α^–C^α^ distance of 5.4 Å; shortest distance of 2.9 Å), I125C (C^α^–C^α^ distance of 5.3 Å; shortest distance of 3.9 Å), D126C (C^α^–C^α^ distance of 3.4 Å; shortest distance of 2.7 Å), and H264C (C^α^–C^α^ distance of 16.8 Å; shortest distance of 10.5 Å). However, only the positions of residues D122C, I125C, and D126C predicted by our model are in agreement with the experimental data. On the other hand, it is worth noting that Leu106, an extracellular loop residue, is anticipated to have considerable flexibility and could feasibly adopt an alternative conformation closer to His_6_ than that predicted by our model, thereby agreeing with the experimental finding.

### Interactions between d-Phe_7_ of the tetrapeptide and MC4R

The d-Phe_7_-to-Cys analogue showed the most visually intense interaction with the I125C mutant when compared to any other of the peptides and to any of the mutant receptors (Fig. 3). It was also shown that peptide 7D interacts with Asp122 and His264. From our model, d-Phe_7_ lies in a pocket surrounded by Phe51 of TM1, Glu100 of TM2, and Ile125, Asp126, and Ile129 of TM3. Hogan *et al.* postulated that Ile125, Ile129, and Ile291 form a hydrophobic pocket with Ile291 on top of the DPxxY motif, possibly acting as a rotamer switch involved in the activation of the receptor, consistent with our model.^23^ The cross-linking studies indicated that the strongest interaction between the peptide and the receptor involved d-Phe_7_ and I125C (C^α^–C^α^ distance of 5.1 Å; shortest distance of 3.6 Å, which is also in agreement with our model). Additionally, the distance from d-Phe to Asp122 (7.0 Å; shortest distance of 6.0 Å) predicted by our model indicated a potential interaction, while the other interactions analyzed were modeled with longer distances and were not predicted to occur [e.g., Leu106 (16.2 Å; shortest distance of 13.9 Å) and His264 (20.6 Å; shortest distance of 17.2 Å)].

An examination of the peptide agonists (α-MSH and NDP-MSH) by NMR suggested that l-Phe-containing and d-Phe-containing analogues exist as mirror images of one another.^41^ Lee *et al.* determined from NMR data that α-MSH, which has a His-l-Phe-Arg-Trp core tetrapeptide, adopted a hairpin–loop conformation, while NDP-MSH, which has a His-d-Phe-Arg-Trp core, contained a β-turn instead.^19^ The different orientation that d-Phe (compared with l-Phe) would experience inside the binding pocket may be the reason for the greater potency and longer activity of NDP-MSH relative to α-MSH. Additionally, it was observed that the W258A mutation had a larger effect on the binding affinity and potency of α-MSH relative to NDP-MSH, suggesting that l-Phe is positioned closer to TM6 and fits into the hydrophobic pocket.^34^ However, it is important to note that peptide 7L (l-Phe_7_) showed no cross-linking to any of the MC4R mutants and, hence, while it is likely to be positioned farther away from TM3 towards TM6, no cross-linking was observed with Cys at the His264 position of TM6.

### Interactions between Arg_8_ of the tetrapeptide and MC4R

The cross-linking study indicates that the mutant D122C receptor interacts strongly with peptide 8 (Arg_8_), providing further evidence^34^ that there is an electrostatic interaction between the negatively charged amino acid on the MC4R and the positively charged Arg_8_ on the peptide agonist (Fig. 3). Not only is Arg_8_ shown to interact with Asp122, but an interaction is also formed with Asp126 (Fig. 3). This evidence is consistent with previous findings where exposed Cys130 is modified by (2-aminoethyl)methanethiosulfonate, highlighting the necessity of Asp126 on TM3 for ligand binding.^62^ Our model suggests that Arg_8_ of NDP-MSH can form a direct contact with Asp126 (C^α^–C^α^ distance of 6.52 Å; shortest distance of 5.2 Å). Furthermore, our model suggests that the side chain of Arg_8_ forms a hydrogen bond with Asp122 of TM3 and a backbone interaction with Tyr287 of TM7. Although Tyr287 was not tested experimentally, Hogan *et al.* also postulated that Tyr287 was located in the MC4R binding site and was likely to have direct contacts with MC4R agonists.^23^

### Interactions between Trp_9_ of the tetrapeptide and MC4R

Our cross-linking studies have shown that Trp_9_ was in close proximity to TM6 and TM7 (residues His264 and Met292, respectively) and also suggested an interaction between Trp_9_ and Ile125. The modeling work suggested that the Trp_9_ residue lies in a pocket with d-Phe, Asp126, Ile129, Cys130, and Leu133 of TM3, and with Phe261 of TM6. Reasonably strong interactions of the peptide were observed with H264C (His264 C^α^–C^α^ distance of 11.7 Å; shortest distance of 7.7 Å) and M292C (M292 C^α^–C^α^ distance of 12.6 Å; closest contact of 6.5 Å). In the cross-linking study, however, no direct interactions are predicted in the model, and some structural flexibility should be incorporated as the receptor accommodates binding of the ligand. Previous modeling studies also suggested that the side chain of Trp_9_ may interact with TM6 in a similar region as l-Phe_7_.^13^

### Identification of peptide contacts between the Cys-containing peptide analogues and WT MC4R

**Fig. 4:**
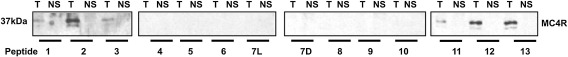
Detection of WT MC4R cross-linked with the cysteine-substituted peptide analogues. Membrane samples prepared from HEK293 cells expressing the hMC4R were incubated separately with 100 nM of each biotinylated cysteine-substituted NDP α-MSH peptide in the absence (T, total binding) or in the presence  (NS, nonspecific binding) of 50 μM SHU9119. Peptide 1 has cysteine at position 1 of the ligand; peptide 2 has cysteine at position 2, and so forth. Peptide 7L has l-cysteine at position 7, whereas peptide 7D has d-Cys at the position. Cys-to-Cys cross-linking was induced by further incubation in the presence of CuP. Samples were then analyzed by SDS-PAGE, followed by Western blot analysis and detection of biotin using streptavidin polyperoxidase and chemiluminescence.

Experiments were performed to cross-link the NDP-MSH Cys analogues to the WT receptor. Figure 4 illustrates the cross-linking of peptide analogues to the native receptor when Cys is present near the amino-terminus of the peptide (particularly at position 2) or near the carboxyl-terminus of the peptide (positions 12 and 13). These data suggested that one or more Cys residues in the WT receptor (there are 15 Cys residues in MC4R) make a direct contact with residues close to the amino-terminus and carboxyl-terminus of the agonist NDP-MSH. The *K*_d_ values for cysteine substitutions at positions 1, 2, 10, and 11 were not significantly different from the native peptide (Student's *t* test, *p* > 0.05; data not shown). In contrast, binding affinity was not readily detectable with cysteine substitutions at positions His-Phe-Arg-Trp (unpublished data).

### Mapping the N-terminus/C-terminus interactions of NDP-MSH and MC4R

A selection of the 15 Cys residues in the MC4R were individually mutated to Ala. These residues were chosen based originally on a bovine-rhodopsin-derived MC4R model.^62^ Seven of these MC4R residues (C40, C130, C177, C196, C257, C271, and C277) occupied positions that are likely to be accessible to the ligand. Peptides 2 and 13 were cross-linked to each Cys-to-Ala mutant receptor to identify the contact site for the N-terminus and the C-terminus of NDP-MSH. Functional characterization of these mutants is presented by Cox *et al.*^62^

**Fig. 5:**
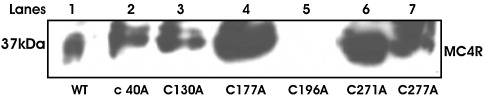
Detection of residue on the MC4R that interacts with the N-terminus of the agonist. Membrane samples prepared from HEK293 cells expressing the MC4R were incubated separately with 100 nM of each biotinylated cysteine-substituted NDP-MSH peptide 2 (T, total binding). The WT receptor (lane 1) was also incubated in the presence of 50 μM SHU9119 (NS, nonspecific binding) and 1 mM GTPγS. Peptide 2 has cysteine at position 2 of the NDP-MSH ligand. Samples were then analyzed by SDS-PAGE, followed by Western blot analysis and detection of biotin using streptavidin polyperoxidase and chemiluminescence. The absence of cross-linking is an indication of the position of interaction of peptide 2 and is located at C196 (lane 6).

**Fig. 6:**
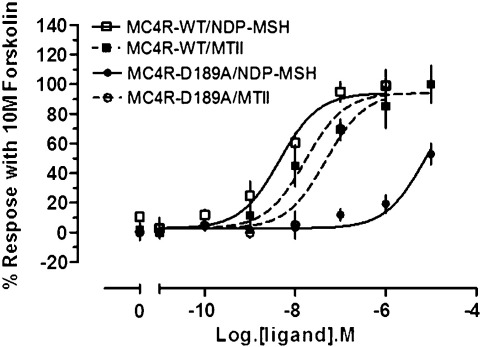
Effect of the D189A mutation of the MC4R on MTII and NDP-MSH agonist stimulation. Activity studies of the MC4R were performed in HEK293 cells stably expressing CRE-Luciferase and with transient transfection of the MC4R. Activity is expressed as a percentage of the luminescence response compared to 10 mM forskolin (a direct adenylate cyclase stimulator). The development of the CRE-Luciferase assay was described by Stables *et al.*^78^ Twenty-four hours posttransfection, HEK293 CRE-Luciferase cells were plated and cultured for a further 12–18 h. The medium was replaced, and the appropriate concentration of the agonist NDP-MSH was added. After incubation, Luclite® reagent (Perkin-Elmer) containing the substrate for the luciferase was added to each well. Luciferase activity was determined by scintillation counting. The data are representative of at least three independent experiments performed in triplicate and analyzed in triplicate by one-site competition plots using GraphPad PRISM 3.02 software. NDP-MSH pEC_50_ values were shifted from 8.27 ± 0.27 to 5.15 ± 0.12 (over 1000-fold) when D189 was mutated to Ala, respectively; however, only a small shift from 7.78 ± 0.13 to 7.33 ± 0.18 (less than 3-fold) was seen for the truncated agonist MTII.

Cross-linking was not observed between the mutant receptor C196A and peptide 2 (Fig. 5), implying that Cys196 contained within the receptor is located in close proximity to residue Tyr_2_ on NDP-MSH. Residue Cys196 is predicted to be located at the extracellular end of TM5 and is orientated into the binding pocket. Previous data have suggested that substitution at positions Ser_1_, Tyr_2_, and Ser_3_ in NDP-MSH, with less hydrophilic residues, slightly reduces affinity and potency.^63,64^ By docking the d-Phe_7_-Arg_8_ β-turn form of the peptide into our MC4R model, we observed interactions between ECL2 and TM5, consistent with the biochemical data. From our model, Ser_1_ of the peptide ligand is predicted to form hydrogen bonds with Val179 on TM4 of the receptor. Our MC4R model also predicted another polar residue located on TM5 (Ser188) to be involved in hydrogen bonding to the N-terminus of the peptide. Although the C196A mutation of the MC4R showed little effect on ligand binding,^62^ when the MC4R was mutated to D189A (ECL2), a dramatic decrease in both affinity for the agonist NDP-MSH (no binding detected) and potency of the agonist NDP-MSH (from pEC_50 _= 8.27 ± 0.27 to pEC_50_ = 5.15 ± 0.12; WT *versus* D189A; over 1000-fold) was observed (Fig. 6). Interestingly, with the truncated cyclic peptide agonist analogue MTII, efficacy did not appear to be affected to the same extent (from pEC_50_ = 7.78 ± 0.13 to pEC_50_ = 7.33 ± 0.18; under 3-fold).

**Fig. 7:**
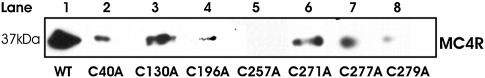
Detection of residue on the MC4R that interacts with the C-terminus of the agonist. Membrane samples prepared from HEK293 cells expressing the MC4R were incubated separately with 100 nM of each biotinylated cysteine-substituted NDP-MSH peptide 13 in the absence (T, total binding) or in the presence  (N, nonspecific binding) of 50 μM SHU9119 and 1 mM GTPγS. Peptide 13 has cysteine at position 13 of the NDP-MSH ligand. Cysteine-to-cysteine cross-linking was induced by further incubation in the presence of CuP. Samples were then analyzed by SDS-PAGE, followed by Western blot analysis and detection of biotin using streptavidin polyperoxidase and chemiluminescence. The absence of cross-linking is an indication of the position of interaction of peptide 13. Molecular mass is expressed in kilodaltons. The MC4R/peptide complex is located at 37 kDa.

In the model, the C-terminus of the peptide ligand is close to the extracellular end of TM6 and TM7, and ECL3 (Fig. 7). Western blot analysis of peptide 13 demonstrates cross-linking of the peptide to all of the mutant receptors, except for C257A-MC4R, implying that the carboxyl-terminus interacts with TM6 (Fig. 3). The Cys(6.47) residue is located on TM6 of the MC4R (Cys257) just below the hydrophobic pocket (Trp257, Phe261, Tyr268, and Ile269). It is conceivable that due to TM6 flexibility, Cys257 in the MC4R may be more accessible in the active state of the receptor.

## Discussion

The aim of this work was to better map the amino acid residues surrounding the binding pocket of the MC4R for one of its agonists, NDP-MSH, using a covalent attachment approach, coupled with a more accurate modeling of the receptor in its activated state. In order to identify specific interactions between the MC4R and NDP-MSH, we cross-linked cysteine-containing peptide analogues to the endogenous and mutated Cys residues on the receptor. The peptide ligands were labeled with biotin at their N-terminus and cross-linked to the receptor, and the complex was detected using streptavidin polyperoxidase.

In this study, we have demonstrated direct interactions between Cys-containing ligand homologues and both the WT receptor and versions containing substituted cysteines, which resulted in receptors to which the peptide ligand was covalently attached. The results revealed the proximity of residues at positions 5–9 of NDP-MSH to residues Leu106, Asp122, Ile125, Asp126, His264, and Met292 of the MC4R. Each position on the core peptide was shown to interact to varying degrees with at least two of the aforementioned residues on TM3, TM6, and TM7. However, a certain amount of caution should be exercised when analyzing the cross-linking data. There is the possibility of interpreting interactions that are not “real” based on a low level of cross-linking due to the potential of trapping serendipitous interactions (e.g., as the peptide enters the binding cleft) or flexibility (as the receptor and ligand “accommodate” the interaction). Furthermore, when charged residues either on the ligand or on the peptide are substituted with cysteines, important electrostatic interactions between the ligand and the receptor may be affected.

To aid the interpretation of these binding data, we constructed a theoretical model whereby the core tetrapeptide was docked initially, followed in a sequential manner by the N-terminus and the C-terminus of the peptide, akin to the proposed binding mode of adrenaline to the β_2_-adrenergic receptor.^65^ A number of research groups have developed R^⁎^-state models for the MC4R, including Schiöth *et al.*, who utilized an MC4R molecular model to suggest that a 76° counterclockwise turn of TM3 may be important for MC4R activation.^41^ Pogozheva *et al.*^33^ employed two different structural templates, namely, a model of the inactive conformation of the hMC4R^29^ and a model of the active conformation of the μ-opioid receptor. They then used distance constraints from the inactive conformation of rhodopsin, together with experimental constraints compatible with active states in several GPCRs, to develop an R^⁎^ model of the MC4R into which they positioned NDP-MSH into the binding pocket using distance geometry calculations. Cho *et al.*^20^ used Autodock^66^ to position an NMR structure of NDP-MSH in the binding pocket of their MC4R model, followed by refinement with a short molecular dynamics simulation. They determined that NDP-MSH formed a β-turn conformation around the d-Phe_7_-Arg_8_ sequence, with the hydrophobic side chain of the d-Phe_7_ residue located away from the negatively charged side chains of the acidic residues. In their model, the Arg_8_ residue is involved in charge–charge interactions with the acidic residues. Finally, Hogan *et al.*^23^ used the observation that a switch in the orientation of Trp258 (from perpendicular to the plane of the membrane to parallel with the plane of the membrane)^49^ and a change in the conformation of the aromatic cluster of residues in TM6 are both involved in receptor activation.^67^ In developing their MC4R R^⁎^ model, Hogan *et al.* first changed the conformation of Phe254, Trp258, and Phe262 in their bovine-rhodopsin-based MC4R model, and then reduced the kink induced by Pro260 in TM6 from an initial 30° to a final lower kink of 11°.^23^

Here, we utilized the recently elucidated crystal structures to generate R^⁎^-state models of the MC4R, from which the structure of a complex with NDP-MSH was developed in a ligand-based modeling approach. This approach allowed us to examine the proposed interactions and the roles of the various mutations studied here and reported earlier in the literature. Previous NMR studies on isolated NDP-MSH have resolved two different structures for the peptide ligand. Cho *et al.* indicated that the central residues forming the turn conformation in NDP-MSH are the His_6_-d-Phe_7_ pair.^20^ In contrast, Hogan *et al.* later studied cyclic decapeptides with the consensus tetrapeptide sequence His-d-Phe_7_-Arg_8_-Trp locked in a type II′ β-turn.^23^ With the use of our MC4R R^⁎^-state model based on the opsin template, the d-Phe_7_-Arg_8_ turn structure fitted the biochemical data best. On the other hand, some of the biochemical data did fit both models; however, it may be that different conformational forms of the ligand interact preferentially with different conformational states of the receptor. Our model is, however, limited by focusing on the predicted active state based on the active state of opsin.^51^

It appears that agonist binding may occur in at least two stages. Firstly, the core tetrapeptide is predicted to neutralize the negative repulsion between TM2 and TM3. The documented interaction between Arg_8_ on the peptide and Asp122 on the receptor^34^ was in agreement with our data, which also indicated that Asp126 is within interaction distance.^62^ In this study, as supported by both cross-linking studies and modeling analysis, both Glu_5_ and His_6_ are shown to be in close proximity to TM3, including the residues Asp122, Asp126, and Ile125 (see Supplementary Material). His_6_ in the core tetrapeptide is not predicted to form direct interactions with the acidic bundle on TM3 of the MC4R, but maintains the “bioactive” conformation of the peptide ligand.^32,68–70^ Some data, however, have implicated residues Asp122, Asp126, His264, and Phe218^32,34^ in forming direct interactions with His_6_ (Supplementary Material). Here, our cross-linking studies and model suggest that His_6_ lies in close proximity to Asp122 and Asp126, but does not interact with the receptor. Rather, His_6_ forms interactions with residues of the peptide ligand itself, including d-Phe_7_, Arg_8_, and Trp_9_, thus supporting the theory of the importance of His_6_ in stabilizing the structure of the peptide ligand.^22,32,68–70^

Previous data have highlighted the importance of burying the hydrophobic residues (d-Phe_7_ and Trp_9_) in the hydrophobic cleft on TM6. Interestingly, one of the strongest cross-links detected was that between Ile125 (TM3) and d-Phe_7_ on the peptide. This interaction was pivotal in our decision to use the d-Phe_7_-Arg_8_ β-turn of NDP-MSH over the His_6_-d-Phe_7_ turn for the selection of our model complex. Interactions between TM3 and d-Phe_7_ have only recently been suggested,^23^ implicating a third interaction in addition to the ionic TM3 interaction and TM6 hydrophobic binding pocket. The ionic interactions may allow rotation of TM3 towards TM2, which unlocks the putative activation motif DRY. Interestingly, one might expect the positively charged residue at position 8 (Arg_8_) to be responsible for activation. However, Arg_8_ has been implicated mainly in potency and affinity, while the peptide ligand position 7 (Phe/Nal_7_) is mainly responsible for agonist/antagonist characteristics.^71^ For example, nonpeptide agonists for the MC4R have been developed excluding any involvement of the Arg_8_ moiety.^22^ Furthermore, d-Phe_7_ has been shown to increase the potency of the ligand; some even argue that it interacts more strongly with the receptor than with l-Phe.^7^ In the antagonist, d-Nal may exert steric hindrance, preventing the movement of TM3. The lack of interaction between the l-Phe_7_ peptide analogue and any of the key predicted residues of the receptor may be of some significance, and it may suggest that there is an alternative binding cavity sampled by l-Phe that has not been exploited in our cross-linking study (e.g., with Phe284 and Tyr287 of TM6).

Experimentally, Trp_9_ on the peptide ligand was shown to be in close proximity to both TM6 and TM7, but the modeling data also indicated that residues on TM3, including Asp126, Ile129, Cys130, and Leu133, lie in the proximity of this residue. However, the predicted proximity of Cys130 to Trp_9_ at 5.2 Å in the model is not supported by the cross-linking studies. Perhaps the environment is not conducive to oxidation or the residues are not appropriately positioned.

In contrast, Met292 of TM7 was newly identified to interact with Trp_9_. This interaction is interesting, as the equivalent residue in bovine rhodopsin Lys296 is involved in covalently attaching the chromophore to the receptor. For the first time, this residue has been shown to be located in the NDP-MSH pocket, as demonstrated by cross-linking (Fig. 3) and functional studies (Table 1). In this study, NDP-MSH exhibited the characteristics of the binding of an antagonist to the mutant M292C-MC4R. Furthermore, previous studies demonstrated that M292C-MC4R exerted a dominant-negative effect on activation when in complex with another form of the receptor (unpublished data). Also, a cysteine at the equivalent position in the neurokinin NK2 receptor was shown to be cross-linked to the C-terminal residues of that ligand.^72^ All these reinforce the significance of this residue in the ligand binding cavity of many GPCRs.

The residues His_6_, Arg_8_, and Trp_9_ on the peptide ligand all showed a level of interaction with TM6 at residue His264. This residue in our model does not appear to be directly involved in the active binding pocket, but previous data have implicated it as important for ligand binding.^33,34,40,73^ Nickolls *et al.* calculated a nonsignificant change in affinity for the peptide agonist NDP-MSH with H264A-MC4R.^37^ Here, we show that activity is dramatically reduced when His264 is mutated to Cys. This evidence points toward His264 being involved in the active-state structure of the MC4R. Leu106 is also not predicted to be directly involved in the agonist binding pocket, but cross-linking has shown interactions with residues His_6_ and d-Phe_7_ on the peptide. This residue is located at the top of TM2 on ECL1 in the model and is predicted to interact with the backbone of nearby Ile102 and Leu107. The side chain is exposed to the solvent, however, and, given its loop position, is likely to be flexible and thus could adopt alternative conformations. The residue at this position in the melanocortin-1 receptor (MC1R), when mutated, caused constitutive activity.^36^ This mutation L106C may alter the proposed ionic arrangement between TM2 and TM3, as suggested from studies of the homologous residue in MC1R, thereby resulting in constitutive activity.^36^ A large body of evidence suggests that Arg_8_ is involved in stabilizing the equilibrium between Asp122 (TM3) and Glu100 (TM2).^43^ Thus, if the equilibrium is altered by the mutation L106C, the function of Arg_8_ becomes redundant. This finding is supported by our binding data (Fig. 3) where peptide 8, which contains the replacement of Arg_8_ with Cys_8_ when bound to L106C-MC4R, behaves similarly to the interaction of NDP-MSH with WT MC4R.

After the core tetrapeptide of NDP-MSH had been docked, its N-terminal and C-terminal regions were mapped into the model, and a series of potential hydrogen bonds between TM4, ECL2, and TM5 was apparent, consistent with the biochemical data. Previous data have suggested that substitution at positions Ser_1_, Tyr_2_, and Ser_3_ of NDP-MSH, with less hydrophilic residues, slightly reduces affinity and potency.^63,64^ From our model, Ser_1_ of the peptide ligand is predicted to form a hydrogen bond with Val179 on TM4 of the receptor; thus, a hydrophobic residue at this peptide position can be expected to reduce affinity. Our data and model also validate a novel interaction between Cys196 (TM5) and Asp189 (ECL2) with the N-terminal region of the peptide (see Supplementary Material). Investigation into the region on the MC4R (TM5) where the amino-terminus of the peptide agonist docks raised the possibility of a hydrogen-bond interaction between Ser188 on the receptor and Ser_3_ on the peptide. It is perhaps relevant that mutation of Asp189 to Ala dramatically decreased both the affinity and the potency of NDP-MSH.

It is important to note that all attempts to mutate or extend ECL2 destroyed the functionality of the MC4R (Cox *et al.*, unpublished data). The modeling work suggests that the small ECL2 forms important interactions with the peptide ligand and is clearly very critical.^32^ The binding of the short agonist MTII, which lacks the N-terminal Ser_1_-Tyr_2_-Ser_3_ motif of NDP-MSH activity, is not affected in the D189A mutant. Therefore, the hydrophilic nature of TM4 and TM5 may be important in forming a binding pocket for the amino-terminus of the peptide ligand. Although the N-terminal region of the peptide, when absent, is not required, it may exert an effect on the conformation of the “bioactive” sequence when present. The residue Cys196(5.42) in TM5 of the MC4R is equivalent to Ser204(5.42) in TM5 of the β_2_-adrenergic receptor. This residue is one of a cluster of Ser residues at the TM5/ECL2 interface that are mainly involved in hydrogen bonding to the catecholamine agonist.^74^ These data begin to shed light on the critical nature of ECL2 in WT MC4R.

For the first time, direct interactions of the carboxyl-terminus of NDP-MSH with the MC4R were observed. The residue at position Val_13_ was shown to be in close proximity to Cys257(6.47) located on TM6. Cys257(6.47) is at an interesting position, as the equivalent residue in the β_2_-adrenergic receptor is accessible, but only in the active conformation.^55^ Furthermore, recent studies that engineered metal ion binding sites in the β_2_-adrenergic receptor demonstrated that this region moves towards TM3, in particular towards Asp(2.29), upon activation by an agonist.^75^ It is suggested that this mechanism of action is common to all GPCRs.^76^ The amino-terminus and carboxyl-terminus of the NDP-MSH ligand have previously been implicated in potency.^28^ The docking of the carboxyl-terminus produced here provides tentative support for the suggestion that the MC4R is activated by sequential interactions of the agonist with the receptor.^65^ Subsequent interactions may include possible hydrogen bonds between TM5 and the amino-terminus of the peptide. Once the receptor is activated, the N-terminal and C-terminal regions of the peptide are able to dock at the TM4/TM5 and TM6/TM7 interfaces, respectively, potentiating the signal. If the peptide ligand docks by a series of steps, each exposing more and more interfaces, this would complement experiments performed on the β_2_-adrenergic receptor that led to the proposal of multiple binding states.^65^

## Materials and Methods

### Materials

#### General materials

The expression vector pcDNA3 containing cDNA for the hMC4R was provided by Dr. Sharon C. Chetham (BASF Pharma, Nottingham, UK). [^125^I]NDP-MSH was obtained from Perkin-Elmer Life Sciences (Boston, MA, USA). Nonradioactive peptides were purchased from Bachem (St. Helens, UK), cell culture reagents were obtained from Invitrogen (Paisley, UK), and protease inhibitor cocktail tablets were obtained from Roche (Lewes, UK). Oligonucleotide primers were synthesized by Sigma-Genosys Ltd. (Pamisford, UK).

#### Cysteine-containing peptides

Peptide analogues of NDP-MSH were designed with l-Cys residues substituted into each position and with biotin attached to the amino-terminus. In total, 14 peptides were synthesized, as both the l-Phe form and the d-Phe form of the ligand (α-MSH and NDP-MSH, respectively) are able to interact with the MC4R with nanomolar affinity (Table 2). The peptides were designed, synthesized, purified, and donated by BASF Pharma.

### Mutagenesis

Point mutations were introduced into the MC4R sequence using the QuikChange® Site-Directed Mutagenesis Kit (Stratagene), with pcDNA3-MC4R vector as template.

### Cell growth

HEK293 cells were routinely maintained in Dulbecco's modified Eagle's medium supplemented with 10% fetal calf serum, 100 μl/ml penicillin, 100 μg/ml streptomycin, and GlutaMAX™ (Invitrogen). Stably transfected cells were grown in the same medium supplemented with 800 μg/ml geneticin G418 sulfate. Cells were grown at 37 °C in 5% CO_2_.

### Cross-linking the peptide to the receptor

Membranes were diluted to a concentration of 25 μg/ml with binding buffer [25 mM Hepes–KOH, 1.5 mM CaCl_2_, 1 mM MgSO_4_, 100 mM NaCl, and 1 mM GTPγS (pH 7.0) plus the protease inhibitors]. Each peptide (concentrations are experiment dependent) was added to the membranes and allowed to equilibrate for 45 min. At 22 °C for 1 h, cross-linking was induced by addition of 5 mM CuP. The reaction was stopped by addition of 5× SDS sample buffer [60 mM Tris–HCl (pH 6.8), 2% SDS, and 10% glycerol] containing 25 mM *N*-ethyl maleimide to react with any free sulfhydryl groups. Nonspecific binding was determined by addition of excess SHU9119.

### CRE-Luciferase assay

The development of the CRE-Luciferase assay was described by Stables *et al.*^77^ and has previously been utilized in our laboratory.^78^ Twenty-four hours posttransfection, HEK293 CRE-Luciferase cells were plated onto 96-well black-wall, clear-bottom, poly-d-lysine-coated microplates (Corning Costar) at a density of 50,000 cells/well and cultured for a further 12–18 h. Medium was replaced with 100 ml of Phenol-red-free culture medium, including IBMX and the appropriate concentration of the agonist NDP-MSH. Microtiter plates were incubated for 4 h at 37 °C, after which Luclite® reagent (Perkin-Elmer) was added to each well. The plates were sealed and subjected to dark adaptation for 5  min at room temperature. Luciferase activity was determined by scintillation counting using a TOPCount scintillation counter (Perkin-Elmer).

### Data analysis

All measurements, except where stated, were carried out in triplicate in three independent experiments. The values quoted and depicted graphically are the means of independent determinations with the standard error of the mean. In competition binding studies, counts were normalized to the maximal specific binding within each data set, and IC_50_ values were calculated with a single site-binding model fitted with the aid of GraphPad PRISM 3.0 software (San Diego, CA). *K*_d_ values were obtained from the IC_50_ using the following equation: *K*_d_ = IC_50_ − [radioligand].^79^ In a similar fashion, EC_50_ values were calculated from the fit of sigmoidal concentration–response curves (three-parameter fit). pIC_50_ refers to − log IC_50_, and pEC_50_ refers to −  log EC_50_. The significance of differences between values was determined by a comparison of the mean values using two-tailed unpaired or paired Student's *t* test.

#### Comparative modeling of MC4R: Sequence alignment

Recently, crystal structures of the active GPCR opsin in an R⁎ state (PDB code 3CAP; 2.9 Å)^50^ and of a complex derived from the C-terminus of the Gα subunit of transducin (PDB code 3DQB; 3.2 Å),^51^ which have previously been used for modeling the active state hβ2R, were published.^80^ The global sequence identity between the MC4R and opsin is only ∼ 16%, which is generally considered not to be sufficient for reliable homology modeling.^81,82^ However, when the TM regions are considered, the identity increases: TM1, 13.3% (43.3%); TM2, 26.7% (60%); TM3, 18.2% (42.4%); TM4, 4.3% (34.8%); TM5, 25% (50%); TM6, 16.1% (58.1%); TM7, 14.3% (52.4%).^83^

A multiple-sequence alignment was made between the amino acid sequence of the opsin and the MC4R (SwissProt accession code P32245) using CLUSTAL W^84^ and subjected to manual inspection to confirm that the conserved residues among most GPCRs^85^ were aligned (496 class A sequences). The alignment was in agreement with that published by Yang *et al.*^83^ However, α-aneurysms (the insertion of an extra residue in a turn) are present in TM2 and TM5 of rhodopsin, but may be absent in other GPCRs, with such a misalignment in the area around such insertions being potentially detrimental to modeling. Chai *et al.*^29^ utilized an alternative alignment for TM2 of the MC4R with a gap being aligned to Gly89 of rhodopsin, resulting in the conserved Glu100 in TM2, which, being oriented into the binding pocket, has been thought to be important for ligand binding.^34^ A similar orientation of Glu100 is also proposed by Haskell-Luevano *et al.*^35^ In contrast, Chai *et al.* determined that Met200 (hMC4R) in TM5 was orientated into the pocket when the rhodopsin α-aneurysm was preserved.^29^ In this work, an additional set of models was generated using the alignment of Chai *et al.*^29^

#### MC4R disulfide bonds

The melanocortin family is interesting as it lacks the family A disulfide bond between TM3 and ECL2. Instead, there is a putative disulfide bond in ECL3 between Cys267 and Cys275 in the MC1R.^86^ In the MC4R, there may be a similar bond between Cys277 and Cys279; in the absence of one of these Cys residues, the other is induced to form a deleterious disulfide bond with Cys271.^87^ In contrast, in our hands (Dr A. Cox, unpublished data), similar mutants do not show the same effect. It is possible that previous studies^87^ reflect more the nature of the substituted residue than disulfide bond formation: Cys-to-Ala mutations in our case *versus* Cys-to-Arg mutations in Tarnow *et al.*^87^ This is reinforced by our SDS-PAGE analysis in the absence of β-mercaptoethanol, where no change in migration was observed. Subsequently, no disulfide bonds were enforced in the modeling work.

#### Models of the R⁎-state MC4R

With the above alignments and individual and template combinations, we used Modeller9v5^61^ to generate different models of the MC4R. The Modeller software implemented comparative protein structure modeling by satisfying spatial restraints.^61^ The alignment is used to construct a set of geometrical criteria that are converted into probability density functions (PDFs) for each restraint. The PDFs include the following:

(1) homology-derived restraints on distances and dihedral angles in the target sequence, taken from its alignment with the template structure(s)
(2) stereochemical restraints such as bond length and bond angle preferences, obtained from the CHARMM-22 molecular mechanics force field
(3) statistical preferences for dihedral angles and nonbonded interatomic distances, obtained from a representative set of known protein structures.^88^

PDFs restrain C^α^–C^α^ distances, main-chain N–O distances, and main-chain–side-chain dihedral angles. The three-dimensional model of a protein is obtained by optimization of molecular PDFs such that the model violates the input restraints as little as possible. Four hundred models of the backbone of the target complex were developed, and a global optimization procedure refines the positions of all heavy atoms in the protein. A subsequent simulated annealing refinement protocol was applied to the loop regions.^89^ The best model was selected using a combination of the Modeller objective score and a selection of protein assessment tools. PROCHECK^90^ was employed to perform a stereochemical check, with every amino acid being classified as having a favored, additionally allowed, generously allowed, or disallowed conformation. ERRAT^52^ counts the number of nonbonded interactions between atoms (CC, CN, CO, NN, NO, and OO) within a cutoff distance of 3.5 Å and yields an overall quality factor for each structure, which is expressed as the percentage of protein for which the calculated error value falls below a 95% rejection limit. Normally accepted model structures produce values above 50, with a higher score indicating that the model has a better ratio of nonbonded interactions. The final model selected yielded the overall best performance across the validation tools, coupled with a structural analysis of the binding pocket.

#### Models of the MC4R/peptide β-turn

d-Phe_7_-Arg_8_-Trp_9_ was previously determined as the minimal NDP-MSH fragment that possesses full agonist efficacy.^34^ The triplet peptide RFF, the key motif of the natural antagonist AGRP, was extracted from the NMR structure (PDB code 1HYK) and used to model the core peptide of NDP-MSH, Glu_5_-His_6_-d-Phe_7_-Arg_8_-Trp_9_, as a β-turn. Although earlier work suggested that the center of the message sequence (d-Phe_7_-Arg_8_) of NDP-MSH would form a stable turn structure, the NMR work of Cho *et al.* on NDP-MSH indicated that the central residues forming the turn conformation are the His_6_-d-Phe_7_ pair.^20^ In contrast, Hogan *et al.* examined cyclic decapeptides with the consensus tetrapeptide sequence His-d-Phe-Arg-Trp locked in a type II′ β-turn and produced an NMR model with the turn at d-Phe_7_-Arg_8_.^23^ Given this uncertainty, peptide models with the β-turn at His_6_-d-Phe_7_ and d-Phe_7_-Arg_8_ were used in the subsequent studies.

The core peptide was manually docked into the pocket of the MC4R in such a way that Arg_8_ was positioned within interacting distance of a negatively charged pocket consisting of Asp122 and Asp126 of TM3, with Trp_9_ positioned closer to TM6. These “R⁎ models” served as starting structures in an automated docking procedure using Autodock,^91^ allowing for peptide side-chain flexibility. Partial charges on the protein and peptide were determined using Kollman charges. A cubic grid of 70 Å × 70 Å × 68 Å around the active site was constructed using the Autogrid program, with a grid point step of 0.375 Å. A Lamarckian genetic algorithm, coupled with local search, was used for docking, with the default parameters implemented in Autodock4. The number of docking runs was set to 175, while ga_num_evals was set to 2,500,000 and ga_num_generations was set to 27,000. To generate input files, we used the AutoDockTools program†. The conformations showing a lower free energy of binding for each ligand were further analyzed.

#### Models of the MC4R/NDP-MSH complex

To further refine the complex structure, we modeled the full peptide structure complexed with the MC4R model using Modeller9v5 after adapting the CHARMM topology files in Modeller9v5 to accurately structure Nle and d-Phe. In this way, we modeled conformational changes induced by the core peptide first determined as the minimal NDP-MSH fragment that possesses full agonist efficacy at the MC4R,^34^ followed by the fitting of N-terminal and C-terminal regions. The Protonate3D module in MOE^53^ was used to assign optimal free-energy proton geometry and ionization states to the model using a generalized Born electrostatics model. The coordinates of the final model are available upon request.

#### Hydrogen-bond analysis

Hydrogen bonds were enumerated in MOE^53^ and scored by a pairwise comparison of heavy atoms, which includes parameters such as atom types (element, hybridization, and bonding environment), distance, and in-plane and out-of-plane angles of substituents. Having been trained on large quantities of protein data using contact statistics, for some atom pair combinations (e.g., secondary amine nitrogen and carbonyl oxygen) the MOE software considered a hydrogen bond was considered possible and a scoring function was defined.^92^ Receptor residues and ions are included in the two-dimensional interaction plots if they are sufficiently close to the ligand (having defined a maximum distance of 4.5 Å between heavy atoms of the ligand and the receptor ). The distance was then extended to 4.6 Å, within which range a residue must have two atoms, and so on, out to 10 atoms at 5.4 Å.

## Figures and Tables

**Table 1:** WT MC4R and mutant cAMP activity studies on HEK293 CRE-Luciferase cells to determine pEC_50_ values for NDP-MSH and peptide 8 (Arg_8_ replaced with Cys) (*N* = 3)

MC4R	Ligand pEC_50_	
	NDP-MSH	Peptide 8
WT	8.27 ± 0.27	NA
L106C	7.72 ± 0.054	8.19 ± 0.08
D122C	NA	—
I125C	7.98 ± 0.12	NA
D126C	4.57 ± 0.051	NA
H264C	5.67 ± 0.44	NA
M292C	NA	NA

NA, no activity.

**Table 2:**
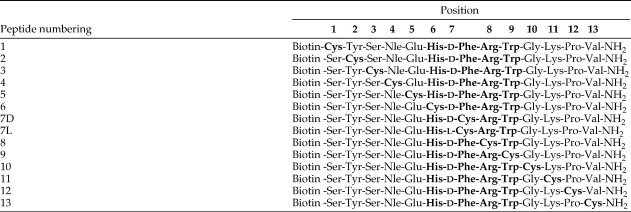
Cys-containing peptide analogues of NDP-MSH

NDP-MSH is made up of 13 amino acids. A Cys residue is replaced in turn for each position in NDP-MSH. Biotin was attached to the amino-terminus.

## Appendix A

Supplementary material The melanocortins α-MSH, β-MSH, and γ-MSH derived from proopiomelanocortin have a conserved core tetrapeptide sequence His_6_-Phe_7_-Arg_8_-Trp_9_, which is the minimal sequence required for activation of the receptor (see Table 1).

## Footnotes

† http://www.scripps.edu/pub/olson-web/doc/autodock/tools.html
