# Supplementary material for: Interactions of the Melanocortin-4 Receptor with the Peptide Agonist NDP-MSH
Source: J Mol Biol. 2010 Aug 20;401(3):433–50. doi: 10.1016/j.jmb.2010.06.028 (PMC3101337; doi:10.1016/j.jmb.2010.06.028)
Supplement: Supplementary material — The melanocortins α-MSH, β-MSH, and γ-MSH derived from proopiomelanocortin have a conserved core tetrapeptide sequence His6-Phe7-Arg8-Trp9, which is the minimal sequence required for activation of the receptor (see Table 1). [file mmc1.doc]

**SUPPLEMENTARY MATERIAL:**

The melanocortins, α-, β- and γ-MSH, derived from proopiomelanocortin (POMC), have a conserved core tetra-peptide sequence –His^6^-Phe^7^-Arg^8^-Trp^9^- which is the minimal sequence required for activation of the receptor, see Table I.

**Table I :** Amino acid sequence of native and synthetic peptide ligands containing the essential tetra-peptide sequence

| **Name** | **Sequence** |
| --- | --- |
| α-MSH | Ac-Ser-Tyr-Ser-Met-Glu-**His-Phe-Arg-Trp-**Gly-Lys-Pro-Val-NH_2_ |
| NDP-MSH | Ac-Ser-Tyr-Ser-Nle-Glu-**His-D-Phe-Arg-Trp**-Gly-Lys-Pro-Val-NH_2_ |
| β-MSH | Ac-Ala-Glu-Lys-Lys-Asp-Glu-Gly-Pro-Tyr-Arg-Met-Glu-**His-Phe-Arg-Trp-**Gly-Ser-Pro-Pro-Lys-Asp- NH_2_ |
| γ-MSH | Ac-Val-Met-Gly-**His-Phe-Arg-Trp**-Asp-Arg-Phe-Gly-NH_2_ |
| MTII | Ac-Nle-c[Asp-**His-D-Phe-Arg-Trp**-Lys]-NH_2_ |
| SHU9119 | Ac-Nle-c[Asp-**His-D-Nal-Arg-Trp**-Lys]-NH_2_ |
| HS014 | Ac-Cys-c[Glu-**His-D-Nal-Arg-Trp**-Gly-Cys-Pro]Pro-Lys-Asp-NH_2_ |
| ACTH | Ac-Ser-Tyr-Ser-Met-Glu**-His-Phe-Arg-Trp**-Gly-Lys-Pro-Val-Gly-Lys-Lys-Arg-Arg-Pro-Val-Lys-Val-Tyr-Pro-Asn-Gly-Ala-Glu-Asp-Glu-Ser-Ala-Glu-Ala-Phe-Pro-Leu-Glu-Phe-NH_2_ |

Table II: Summary of predicted and identified interactions made between residues on the NDP-MSH peptide and the MC_4_R. The most defined interaction and highlighted in bold. N/D not determined. Bold indicates biochemical and modelling data. * indicates a novel interaction. § indicates a peptide-peptide interaction. Italics show the weaker interactions.

| **Residue on NDP-MSH** | **Predicted interaction on MC_4_R** | **Bio chemically Identified interaction on MC_4_R** | **Model proximity based of His^6^-DPhe^7^ turn** | **Model proximity based on DPhe^7^-Arg^8^ turn** |
| --- | --- | --- | --- | --- |
| **Ser 1** |  |  | Phe184, Ile186 of ECL2 and Phe117 of ECL1. | 2.7Å Val179 (TM4) |
| **Tyr^2^** | N/D | Cys196 (TM5)  Asp189 (ECL2) | **7.6Å from Asp189 (ECL2)***  **7.7Å from Cys196 (TM5)*** | Ser^3^ and Lys^11^§  **6.6Å Asp189 ( ECL2**)*  **7.7 Å Cys196 (TM5)*** |
| **Ser 3** |  |  | **Stabilises Peptide** | Lys^11^ and Tyr^2^§  2.8Å Ser188 (ECL2)* |
| **Nle^4^** |  |  | **No contact** | **No contact** |
| **Glu^5^** | N/D | Asp126 (TM3)  *Asp122 (TM3)*  *Ile125 (TM3)* | **9.6Å Asp126 (TM3)***  His264 (TM4)  Cys277(ECL3)  Leu288 (TM7) | 2.7Å Ser127 (TM3)*  **2.6Å Asp126***  **2.5Å Asp122***  ***6.1Å Ile125**** |
| **His^6^** | Asp122/Asp126 (TM3)  His264 (TM6)  Phe284 (TM7)  Ref 1-2 | Leu106 (ECL1)  Asp122 (TM3)  Asp126 (TM3)  His264 (TM6) | **17.6Å Leu106 (ECL1)**  **10.2Å Asp122 (TM3)**  11.0Å Ile125 (TM3)  **8.8Å Asp126 (TM3)**  **12.9Å His264 (TM6)**  Phe51 (TM1)  Ile104 (ECL1)  Glu100 ( TM2)  Phe284 and Leu288 (TM7) | Arg^8^ and Trp^9^ §  **16.5 Å L106C (ECL1)**  **2.9Å Asp122 (TM3)**  **3.9Å Ile125 (TM3)***  **2.7Å Asp126 (TM3)**  **10.5Å His264 (TM6)** |
| **DPhe^7^** | Glu100 (ECL1)  Ile125, Ser127, Ile129, Leu133 (TM3), Phe261 (TM6), Phe284 and Ile291 (TM7)  Ref 3-5 | Ile125 (TM3)  *Leu106 (ECL1)*  *Asp122 (TM3*  *His264 (TM6)*  *Met292 (TM7*) | Glu^5^ §  **11.9Å Ile125 (TM3)**  ***10.6Å His264 (TM6*)**  Ile129 (TM3)  Leu133 (TM3)  Ile291 (TM7) | **3.6Å Ile125 (TM3)**  ***6.0Å Asp122 (TM3)***  ***11.2Å His264 (TM6).***  ***13.9 Å Leu106 (ECL1)***  Phe51 (N-Terminus)*  Glu100 (ECL1)*  Asp216 (TM5)*  Ile129 (TM3)* |
| **Arg^8^** | Asp122 and Asp126 (TM3)  Ref 6 | Asp122 (TM3)  Asp126 (TM3)  *His264 (TM6)* | Glu^5^ and Ser^3^ §  **2.7Å Asp122 (TM3) HB**  **2.7Å Asp126 (TM3) HB** | His^6^ §  **2.9Å Asp122 (TM3) HB**  **5.2Å Asp126 (TM3) HB**  2.8Å Tyr287 (TM7) HB* |
| **Trp^9^** | Ile 129 and Leu 133 (TM3)  Phe184 (TM4), Phe214 (TM5) and Trp257, Tyr268, Ile269 and Ile259 (TM6)  Ref 4-8 | His264 (TM6)  Met292 (TM7)  *Ile125 (TM3)* | Ser^3^ and Glu^5^ §  **7.8Å His264 (TM6)**  **14.7Å Met292 (TM6)**  Phe261 (TM6)  Leu265(TM6)  His264(TM6)  Phe184 (TM5)  Val193 (TM5)  Cys196 (TM5) | His^6^ and DPhe^77^§  **7.7 Å His264 (TM6**)  **6.5Å Met292 (TM7)***  ***5.9Å Ile125 (TM3)***  Asp126 (TM3)  Ile129 (TM3)  Leu133 (TM3)  Phe261 (TM6) |
| Gly^10^ |  |  | No interactions detected | No interactions detected |
| **Lys11** |  |  | Val^13^§  2.0Å Phe267 (TM6)  1.9Å Cys271 (ECL3) | Ser^3^ and Tyr^2^ § |
| Pro12 |  |  | No interactions detected | No interactions detected |
| **C-terminus (Val^13^)** | N/D | **Cys257** (TM6) | No interactions detected | No interactions detected |

**Supplementary Material References:**

**1** Yang, Y., Fong, T., Dickinson, C., Mao, C., Li, J., Tota, M., Mosley, R., Van Der Ploeg, L. & Gantz, I. (2000). Molecular determinants of ligand binding to the human melanocortin-4 receptor. *Biochemistry* 39 , 14900-11.

**2** Fleck, B., Chen, C., Yang, W., Huntley, R., Markison, S., Nickolls, S., Foster, A. & Hoare, S. (2005). Molecular interactions of nonpeptide agonists and antagonists with the melanocortin-4 receptor. *Biochemistry* 44 , 14494-508.

**3**  Hogan, K., Peluso, S., Gould, S., Parsons, I., Ryan, D., Wu, L. & Visiers, I. (2006). Mapping the binding site of melanocortin 4 receptor agonists: a hydrophobic pocket formed by I3.28(125), I3.32(129), and I7.42(291) is critical for receptor activation. *J Med Chem* 49 , 911-22.

**4**  Lim, S., Li, S., Lee, C., Yoon, C., Baik, J. & Lee, W. (2001). Minimization of MC1R selectivity by modification of the core structure of alpha-MSH-ND. *Chem Biol* 8 , 857-70.

5 Chen, M., Cai, M., Aprahamian, C., Georgeson, K., Hruby, V., Harmon, C. & Yang, Y. (2007). Contribution of the conserved amino acids of the melanocortin-4 receptor in [corrected] [Nle4,D-Phe7]-alpha-melanocyte-stimulating [corrected] hormone binding and signaling. J Biol Chem 282, 21712-9.

**6**  Cox, A., Donnelly, D., Kaur, M., Cheetham, S., Cockcroft, V. & Findlay, J. (2005). MTSEA prevents ligand binding to the human melanocortin-4 receptor by modification of cysteine 130 in transmembrane helix 3. *FEBS Lett* 579 , 285-91.

**7** Haskell-Luevano, C., Cone, R., Monck, E. & Wan, Y. (2001). Structure activity studies of the melanocortin-4 receptor by in vitro mutagenesis: identification of agouti-related protein (AGRP), melanocortin agonist and synthetic peptide antagonist interaction determinants. *Biochemistry* 40 , 6164-79.

8 Yang, Y., Cai, M., Chen, M., Qu, H., McPherson, D., Hruby, V. & Harmon, C. (2009). Key amino acid residues in the melanocortin-4 receptor for nonpeptide THIQ specific binding and signaling. *Regul Pept* **155**, 46-54.
